# Association between general joint hypermobility and knee, hip, and lumbar spine osteoarthritis by race: a cross-sectional study

**DOI:** 10.1186/s13075-018-1570-7

**Published:** 2018-04-18

**Authors:** Portia P. E. Flowers, Rebecca J. Cleveland, Todd A. Schwartz, Amanda E. Nelson, Virginia B. Kraus, Howard J. Hillstrom, Adam P. Goode, Marian T. Hannan, Jordan B. Renner, Joanne M. Jordan, Yvonne M. Golightly

**Affiliations:** 1 Thurston Arthritis Research Center, University of North Carolina, 3300 Doc J. Thurston Bldg, CB#7280, Chapel Hill, 27599-7280 NC USA; 2 School of Medicine, University of North Carolina, Chapel Hill, 27599 NC USA; 3 Department of Biostatistics, Gillings School of Global Public Health, University of North Carolina, Chapel Hill, 27599 NC USA; 4 School of Nursing, University of North Carolina, Chapel Hill, 27599 NC USA; 5 Department of Medicine, Duke Molecular Physiology Institute, School of Medicine, Duke University, Durham, 27701 NC USA; 6 Motion Analysis Laboratory, Hospital for Special Surgery, New York, 10021 NY USA; 7 Department of Orthopedic Surgery, Duke Clinical Research Institute, School of Medicine, Duke University, Durham, 27708 NC USA; 8 Institute for Aging Research, Hebrew SeniorLife, Boston, 02131 MA USA; 9 Department of Radiology, University of North Carolina, Chapel Hill, 27599 NC USA; 10 Department of Epidemiology, Gillings School of Global Public Health, University of North Carolina, Chapel Hill, 27599 NC USA; 11 Injury Prevention Research Center, University of North Carolina, Chapel Hill, 27599 NC USA

**Keywords:** Osteoarthritis, General joint hypermobility, Cohort, Race, Pain

## Abstract

**Background:**

Osteoarthritis (OA) prevalence differs by race. General joint hypermobility (GJH) may be associated with OA, but differences by race are not known. This community-based study examined the frequency of GJH and its relationship with knee, hip, and lumbar spine OA by race (African American vs. Caucasian).

**Methods:**

Data were from the Johnston County OA project, collected 2003–2010. GJH was defined as Beighton score ≥4. OA symptoms were defined as the presence of pain, aching, or stiffness on most days separately at the knee, hip, and lower back. Radiographic OA (rOA) of the knee or hip was defined as Kellgren-Lawrence grade 2–4. Lumbar spine rOA was disc space narrowing grade ≥1 and osteophyte grade ≥2 in ≥ 1 at the same lumbar level. Lumbar spine facet rOA was present in ≥ 1 lumbar levels. Separate logistic regression models stratified by race were used to examine the association between hypermobility and rOA or OA symptoms at each joint site, adjusting for age, sex, previous joint injury, and body mass index (BMI).

**Results:**

Of 1987 participants, 1/3 were African-American and 2/3 were women (mean age 65 years, mean BMI 31 kg/m^2^). Nearly 8% of Caucasians were hypermobile vs. 5% of African-Americans (*p* = 0.03). Hypermobility was associated with lower back symptoms in Caucasians (adjusted odds ratio (aOR) 1.54, 95% confidence interval (CI) 1.00, 2.39), but not in African-Americans (aOR 0.77, 95% CI 0.34, 1.72). Associations between hypermobility and other knee, hip, or lumbar spine/facet OA variables were not statistically significant.

**Conclusions:**

General joint hypermobility was more common in Caucasians than African-Americans. Although there were no associations between hypermobility and rOA, the association between hypermobility and lower back symptoms may differ by race.

**Electronic supplementary material:**

The online version of this article (10.1186/s13075-018-1570-7) contains supplementary material, which is available to authorized users.

## Background

Osteoarthritis (OA) is a painful and debilitating joint disease and is a leading cause of disability. Although OA is common in all adults, the prevalence and severity of OA in some joints is greater among African-Americans than Caucasians. For example, compared to Caucasians, African-Americans experience a higher rate of progression [1] and higher prevalence of knee OA [2, 3] and have more severe superior hip joint space narrowing and more hip osteophytes than Caucasians [4]. At the lumbar spine, African-Americans also have a higher prevalence of disc space narrowing, vertebral osteophytes, and facet joint OA compared to Caucasians [5]. These observed differences in OA by race may in part be related to underlying biomechanical factors that alter joint mechanics or increase joint stresses, such as obesity or repetitive occupational activities.

General joint hypermobility (GJH), a condition involving abnormally large range of motion in the joints [6], is a biomechanical factor that may contribute to OA and joint pain [7–11]. Prior studies show variation in the relationship of hypermobility and OA or symptoms by joint site [12–14] and across populations [15–17]. For example, in a study of women 50+ years of age in the United Kingdom, hypermobility was associated with knee OA but had an inverse association with hand OA [12]. Hypermobility is more commonly associated with younger populations and women [15], but few studies have examined whether it varies by race. In a study of 81 Caucasian and 45 African-American women, Wood [18] reported that Caucasians were more likely to have hypermobility of the elbow (18% vs. 6%), the proximal interphalangeal (PIP) joints II–IV (90% vs. 74%), and PIP V (46% vs. 36%) when compared to African-Americans. Conversely, African-Americans exhibited hypermobility of the distal interphalangeal joints II–IV (79% v. 88%) and hyperextension of the thumb interphalangeal joints (52% vs. 66%) more frequently than Caucasians. In a study of US military personnel (male and female), Scher and colleagues [19] observed a higher adjusted incidence rate of a GJH syndrome diagnosis code in Caucasians compared to African-Americans (adjusted incidence rate ratio 1.44, 95% confidence interval 1.19, 1.75). Differences between Caucasians and African-Americans in the occurrence of hypermobility, particularly when including lower body joints, have not been examined in a large, community-based older adult population. Furthermore, it is not known whether the relationship between GJH and OA varies by race.

Considering the potential link between hypermobility and OA along with their independent variation by race, the purpose of this cross-sectional study of a large community-based sample was to (1) describe the frequency of GJH by race and (2) examine the relationship between GJH and radiographic OA (rOA) and symptoms consistent with OA at the knee, hip, and lumbar spine by race. We hypothesized that (1) Caucasians would be more likely to have GJH than African-Americans and (2) the relationship between GJH and rOA or OA symptoms would differ between Caucasians and African-Americans.

## Methods

### Study design

Participants were from the Johnston County OA Project, a community-based cohort study of individuals with and without OA [2]. Non-institutionalized Caucasian and African-American residents aged 45 years or older were recruited from six communities within Johnston County, North Carolina. Because the parent study was designed to examine racial differences in OA development and progression longitudinally, African-Americans were oversampled to allow for such comparisons. This study was approved by the Institutional Review Boards of the University of North Carolina School of Medicine and the Centers for Disease Control and Prevention (UNC 14–3219). All participants provided written informed consent prior to data collection.

### Hypermobility

**Fig. 1:**
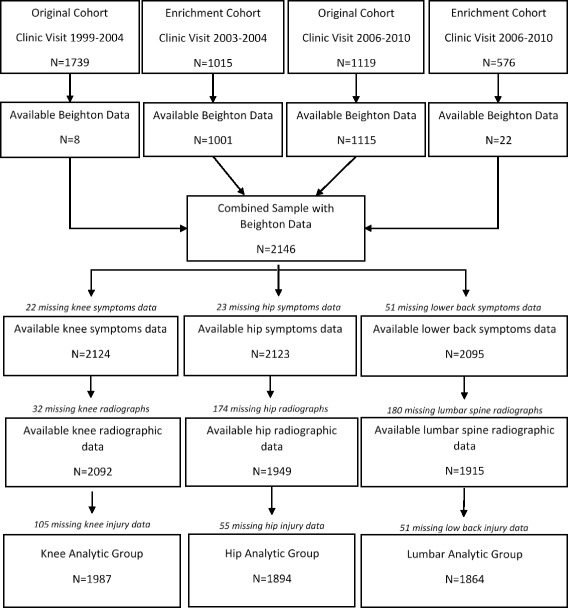
Johnston County participants with OA available for analysis

GJH data were collected during 2003–2010 and were assessed using the Beighton score [20]. For analyses, the first available Beighton score was used. When the Beighton measure was added to the parent project, only eight participants from the original cohort (enrolled 1991–1997) who attended their first follow up clinic visit completed this measure; most original cohort participants who returned for their second follow up visit (2006–2010) completed the Beighton measure (*N* = 1115, Fig. 1). Nearly all of the participants in the enrichment cohort (enrolled in 2003–2004) completed the Beighton measure as part of their baseline clinic visit; 22 enrichment cohort participants completed their first Beighton measure during their first follow up clinic visit (2006–2010, Fig. 1).

For the Beighton criteria, participants were evaluated on their ability to complete nine tasks involving joint range of motion: forward trunk flexion with palms on floor and knees extended, right and left knee hyperextension ≥10°, right and left elbow hyperextension ≥10°, right and left passive dorsiflexion of the 5th finger ≥90°, and right and left passive apposition of thumb to forearm. One point was given for the completion of each maneuver, with scores ranging from 0 (unable to complete any maneuver) to 9 (able to complete all maneuvers). Based on previous literature, general joint hypermobility was defined as a Beighton score ≥4 [6]. Two examiners were trained by the Principal Investigator of the parent study to conduct musculoskeletal assessments and were re-trained prior to follow up data collection of the original cohort and the enrollment or follow up of the enhancement cohort. Inter-rater reliability was high (κ > 0.80) between the two examiners for each of the Beighton maneuvers (see Additional file 1: Table S1).

### Osteoarthritis and symptoms

Knee and hip rOA was defined as a Kellgren Lawrence (KL) grade ≥2. Lumbar spine rOA was defined as disc space narrowing (DSN) grade ≥1 and presence of osteophytes (OST) grade ≥2 at the same lumbar level in at least one lumbar spine level. Lumbar spine facet joint rOA was defined as the presence or absence of OA features (e.g., osseous overgrowth, sclerosis) in at least one lumbar level. Radiographs at each site were evaluated by a single expert radiologist (JBR) who previously conducted intra-rater reliability analysis in a random sample of participants (intra-rater reliability for evaluation of hip/knee [21], κ = 0.89; of DSN [22], κ = 0.89; of OST [22], κ = 0.90; and of facet joints [22], κ = 0.73). Information on the presence of symptoms consistent with OA was obtained via questionnaire with the following question: “On most days, do you have pain, aching, or stiffness in your [right/left] hip/[right/left] knee/lower back?” An affirmative answer determined the presence of symptoms at each particular joint. No time period for the duration of symptoms was specified.

### Covariates

Race, age, and sex were collected via self-report, with race (African-American/Caucasian) and sex (men/women) treated as dichotomous variables. Age and body mass index (BMI; kg/m^2^) were considered as continuous variables. History of joint injury was obtained via questionnaire consisting of two questions: “Has a doctor ever told you that you broke or fractured your [right/left] hip/[right/left] knee/lower back?” and “Other than a fracture, have you injured your [right/left] hip/[right/left] knee/lower back enough to require a cane, cast, or crutch for two weeks or longer?” Injury was defined as a “yes” response to at least one of the questions for a given joint site. BMI was calculated as Weight (kg) / (Height (m))^2^ [2]. Height was measured using a calibrated stadiometer, and weight was measured using a balance-beam scale. Both measures were taken without shoes.

### Statistical analysis

Means and standard deviations for continuous variables, and frequencies and percentages for categorical variables, were calculated for demographic and clinical characteristics at each joint site (knee, hip, and lumbar spine). Differences in percentages of hypermobility, symptoms, and rOA by racial group were assessed using chi-square statistics with a significance level of *p* = 0.05; no adjustments were made to *p* values for multiple comparisons. Separate logistic regression analyses, stratified by race (Caucasian vs. African-American), were carried out to examine the association between hypermobility and symptoms or rOA at the knee, hip, lumbar spine, and lumbar facet joints. Because the occurrence of GJH and OA are known to substantially differ by age, one set of models was adjusted for age only, and another set was adjusted for known OA risk factors including age [23], sex [24], history of joint injury [25, 26], and BMI [27]. All statistical analyses were completed using SAS System Software 9.4 (SAS Institute, Inc., Cary, NC, USA).

## Results

A total of 2146 participants in the Johnston County OA parent study had available Beighton data (1123 participants from the original cohort, and 1023 participants from the enrichment cohort; Fig. 1). For the knee analytic group, 1987 participants were considered for analysis (Fig. 1; Table 1). For the hip analytic group, 1894 participants had available data, and 1864 participants were included in the lumbar spine analytic group (Fig. 1; Table 1). Participant characteristics were generally similar across the three analytic groups; all groups had a mean age of approximately 65 years and mean BMI of 31 kg/m^2^. Nearly two thirds of participants were women, and one third was African-American. In the knee analytic group, 16% of participants had a history of knee injury. Few participants in the hip and lumbar spine analytic groups reported a history of hip (5.0%) and back injury (2.4%). Patient characteristics for all three analytic groups were also similar to the total available participants (*N* = 2146).

**Table 1:** Distribution of GJH and patient characteristics (total sample and by joint site)

	Total sample and analytic groups			
	Total (N = 2146)	Knee (*N* = 1987)	Hip (*N* = 1894)	Lumbar spine (*N* = 1864)
Age (years), mean ± SD	65 ± 11	65 ± 11	66 ± 10	66 ± 10
Women, n (%)	1432 (66.7%)	1322 (66.5%)	1237 (65.3%)	1210 (64.9%)
African-American, n (%)	735 (34.3%)	670 (33.7%)	624 (33.0%)	615 (33.0%)
Body mass index (kg/m^2^), mean ± SD	31 ± 7	31 ± 7	31 ± 7	31 ± 7
Prior joint injury, n (%)	461 (21.5%)	323 (16.3%)	94 (5.0%)	44 (2.4%)

*GJH* general joint hypermobility, *SD* standard deviation

### Frequency of GJH, joint symptoms, and rOA by race

GJH was more common in Caucasians than in African-Americans (7.8% vs. 5.2%, *p* = 0.03; Table 2). Compared to Caucasians, symptoms at the knee occurred more frequently in African-Americans (49.0% vs. 42.6%, *p* < 0.01); a similar but non-significant difference was observed for knee rOA (41.2% vs. 37.2%, *p* = 0.08). Although not statistically significant, hip symptoms and hip rOA were slightly more common in Caucasians than African-Americans (34.3% vs. 30.6%, *p* = 0.11; 34.5% vs. 30.3%, *p* = 0.07, respectively). There was no difference by race in the occurrence of lower back symptoms (40.2% vs. 40.5%), but lumbar spine rOA and lumbar spine facet joint rOA were more common in Caucasians than in African-Americans (61.9% vs. 52.8%, *p* < 0.01; 73.7% vs. 60.5%, *p* < 0.01, respectively).

**Table 2:** Distribution of GJH, joint symptoms and radiographic OA by race, Johnston County OA Project, 2003–2010

	Race		
	Caucasian (*N* = 1317)	African-American (*N* = 670)	*p* value
GJH, n/N (%)	103/1317 (7.8%)	35/670 (5.2%)	0.03
Knee symptoms, n/N (%)	561/1317 (42.6%)	328/670 (49.0%)	<0.01
Knee rOA, n/N (%)	490/1317 (37.2%)	276/670 (41.2%)	0.08
Hip symptoms, n/N (%)	435/1270 (34.3%)	191/624 (30.6%)	0.11
Hip rOA, n/N (%)	438/1270 (34.5%)	189/624 (30.3%)	0.07
Lower back symptoms, n/N (%)	502/1249 (40.2%)	249/615 (40.5%)	0.90
Lumbar spine rOA, n/N (%)	773/1249 (61.9%)	325/615 (52.8%)	<0.01
Lumbar spine facet joint rOA, n/N (%)	937/1272 (73.7%)	370/612 (60.5%)	<0.01

*GJH* general joint hypermobility, *OA* osteoarthritis, *rOA* radiographic osteoarthritis

### Association between GJH and symptoms or rOA by race

As shown in Table 3, there was a positive association between GJH and OA symptoms at the lumbar spine among Caucasians (adjusted odds ratio (aOR) 1.54, 95% confidence interval (CI) 1.00, 2.39), but a non-significant inverse relationship among African-Americans (aOR 0.77, 95% CI 0.34, 1.72). There were no statistically significant associations between hypermobility and knee, hip, lumbar spine rOA, or facet joint rOA for either race group. However, parameter estimates suggested an inverse association of GJH and rOA of the knee, hip, lower back symptoms or lumbar spine rOA and facet joint rOA among African-Americans, while estimates for Caucasians either appeared to be attenuated toward the null (knee rOA, lumbar spine rOA) or suggested a positive association (hip rOA, lumbar spine facet joint rOA).

**Table 3:** Race-specific multivariable logistic regression models for joint symptoms and radiographic OA with GJH

	Beighton ≥4	Beighton <4	Age-adjusted^a^ OR (95% CI)	Multivariably adjusted^b^ OR (95% CI)
Knee symptoms				
Caucasian	42/103 (40.8%)	519/1214 (42.8%)	0.92 (0.61–1.39)	1.00 (0.65–1.56)
African-American	14/35 (40.0%)	314/635 (49.4%)	0.67 (0.33–1.35)	0.70 (0.34–1.47)
Knee rOA				
Caucasian	29/103 (28.2%)	461/1214 (38.0%)	0.77 (0.49–1.23)	0.90 (0.55–1.47)
African-American	9/35 (25.7%)	267/635 (42.0%)	0.45 (0.20–1.02)	0.45 (0.19–1.09)
Hip symptoms				
Caucasian	33/90 (36.7%)	402/1180 (34.1%)	1.13 (0.73–1.77)	1.19 (0.75–1.87)
African-American	9/29 (31.0%)	182/595 (30.6%)	1.02 (0.46–2.29)	0.99 (0.43–2.27)
Hip rOA				
Caucasian	33/90 (36.7%)	405/1180 (34.3%)	1.26 (0.79–1.99)	1.30 (0.82–2.07)
African-American	8/29 (27.6%)	181/595 (30.4%)	0.84 (0.35–2.00)	0.79 (0.33–1.89)
Lower back symptoms				
Caucasian	45/91 (49.5%)	457/1158 (39.5%)	1.50 (0.98–2.30)	**1.54 (1.00–2.39)**
African-American	10/29 (34.5%)	239/586 (40.8%)	0.76 (0.35–1.67)	0.77 (0.34–1.72)
Lumbar Spine rOA				
Caucasian	51/91 (56.0%)	722/1158 (62.3%)	0.77 (0.51–1.18)	0.91 (0.58–1.43)
African-American	12/29 (41.4%)	313/586 (53.4%)	0.62 (0.29–1.31)	0.57 (0.25–1.28)
Lumbar facet rOA				
Caucasian	69/93 (74.2%)	868/1179 (73.6%)	1.03 (0.64–1.67)	1.31 (0.78–2.19)
African-American	16/28 (57.1%)	354/584 (60.6%)	0.87 (0.40–1.86)	0.74 (0.31–1.76)

*OA* osteoarthritis, *GJH* general joint hypermobility, *rOA* radiographic osteoarthritis

^a^Adjusted for age only

^b^Adjusted for age, gender, prior joint injury, and body mass index. Statistically significant odds ratios are represented in bold

## Discussion

To our knowledge, this is the first study to examine GJH and its relationship to OA and OA symptoms among African-Americans and Caucasians in a large community-based sample of middle-to-older-aged adults. Results of this study suggest that the frequency of GJH may be lower in African-Americans than Caucasians and that the link between hypermobility and lower back symptoms may vary by race.

The frequency of GJH in the total sample was modest (around 7%), but Caucasians were somewhat more likely to have GJH than African-Americans, corresponding with previous studies reporting higher frequencies of general hypermobility and hypermobility at the elbow and PIP joints for Caucasians compared to African-Americans [18, 19]. Considering previous reports of African Americans having more severe OA than Caucasians, it is possible that these results may be a reflection of disease severity with subsequent decreases in hypermobility. However, because we do not know the severity of symptoms or radiographic evidence of OA, we are unable to make this determination. In addition, African-Americans had a higher frequency of knee symptoms compared to Caucasians, corresponding with previous literature [2, 28, 29]. However, more Caucasians had rOA in the lumbar region compared to African-Americans; these results agree with a previous study of the Johnston County population, which reported a higher prevalence of lumbar spine disc space narrowing, vertebral osteophytes, and facet joint rOA in Caucasians compared to African-Americans [5].

The present study showed that hypermobility was positively associated with lower back symptoms in Caucasians. Though racial make-up was not reported by Larsson et al. [16] in a study of musicians, a positive association between GJH and lower back symptoms was also reported; specifically, 23% of musicians with hypermobility in the spine (i.e., positive Beighton trunk maneuver) had back symptoms (e.g., pain, weakness, stiffness) compared to 11% without hypermobility (*p* < 0.001). Interestingly, however, when assessing trunk hypermobility in the present study, we found inverse relationships between trunk hypermobility and lower back symptoms in both Caucasians (aOR = 0.37 (0.20–0.68)) and African Americans (aOR = 0.44 (0.20–0.99)) and 22% of individuals with trunk hypermobility had lower back symptoms compared to 41% without trunk hypermobility. With an average age of 65 ± 11 years in the present study compared to 25 ± 9 years in the study by Larsson et al. [16], this discrepancy may be a reflection of joint stiffening with age corresponding to decreasing hypermobility and increasing joint symptoms common to the presence of OA. Though no racial differences are apparent, future studies should further investigate the influence of age on changes in joint mobility and joint symptoms. In our study hypermobility was inversely associated with knee rOA in African-Americans, though it was not statistically significant (aOR = 0.45 (0.19–1.09)) . Again, this may be a reflection of more progressive joint disease and in African Americans compared to Caucasians, making the relationship between hypermobility and OA less apparent. Though it remains unclear why African-Americans with GJH were less likely to have knee rOA than those without hypermobility, this inverse relationship is not unprecedented as shown in previous studies reporting reduced risk of knee rOA in mostly Caucasian postmenopausal women with GJH [14] and inverse associations between hypermobility and knee rOA in a family-based prospective study of participants of mixed African-American and native American ancestry [13].

The present study benefits from the large community-based sample, the inclusion of African-American and Caucasian participants, the presence of participants who were 45 years of age and older to allow examination of OA, and the detailed data on GJH, rOA, and joint symptoms. However, there were several limitations of this study. These analyses were cross-sectional, and consequently, the contribution of GJH to rOA and progression of joint symptoms is not known. The Beighton criteria provide a general assessment of overall hypermobility, and results may differ if one examines hypermobility and rOA or symptoms at a single joint site (e.g., trunk hypermobility and lower back symptoms). In addition, this study assessed current hypermobility (i.e., at the time of the study visit) in our older adult sample. GJH is a condition found more commonly in younger individuals, potentially contributing to challenges with identifying relationships between hypermobility (waning with age as joints tend to stiffen over time [30, 31]) and OA (increasing with age). Thus, the lack of statistically significant associations between hypermobility and joint symptoms and rOA in our study may reflect the older age of our population. Studies that assess hypermobility in youth and subsequent rOA or joint symptom development and/or progression would clarify this association. The injury variables used as covariates in these analyses were based on self-report, and there may be recall bias in which those with rOA or joint symptoms were more likely to recall a prior injury than those who were asymptomatic or free of joint disease. However, the use of medical records to assess history of injury may not be reliable either, since not all joint injuries are reported to health professionals (particularly if the individual considers the injury to be minor or recurrent), and retrospective retrieval would be difficult for those who had sustained an injury several decades prior to study assessments. The benefit of self-report over medical records is that it may capture more occurrences of injury.

## Conclusions

In conclusion, Caucasians had a somewhat higher prevalence of GJH than African-Americans, although it was not a common condition in either group in this older adult population. Although our study results should be interpreted with caution, patterns suggest that associations of GJH and lower body OA symptoms may differ by race. Caucasians with hypermobility were more likely to have lumbar symptoms than those without hypermobility, but similar associations were not observed in African-Americans. Longitudinal studies of the changing relationship between hypermobility and rOA or OA symptom progression can further advance our understanding of the roles of hypermobility and race in OA.

## Additional file

Additional file 1: **Table S1.** Inter-rater reliability (κ) and 95% confidence intervals of Beighton scores at individual sites. Inter-rater reliability of two examiners conducting the Beighton measure. (DOCX 12 kb)

## Abbreviations

aOR: Adjusted odds ratio
BMI: Body mass index
DSN: Disc space narrowing
GJH: General joint hypermobility
KL: Kellgren Lawrence
OA: Osteoarthritis
OST: Osteophytes
PIP: Proximal interphalangeal
rOA: Radiographic OA
