# Supplementary material for: Association between general joint hypermobility and knee, hip, and lumbar spine osteoarthritis by race: a cross-sectional study
Source: Arthritis Res Ther. 2018 Apr 18;20:76. doi: 10.1186/s13075-018-1570-7 (PMC5907300; doi:10.1186/s13075-018-1570-7)
Supplement: Supplementary file 1 — Table S1. Inter-rater reliability (κ) and 95% confidence intervals of Beighton scores at individual sites. Inter-rater reliability of two examiners conducting the Beighton measure. (DOCX 12 kb) [file 13075_2018_1570_MOESM1_ESM.docx]

| **Table S1. Inter-rater reliability (**κ**) and 95% Confidence Intervals of Beighton scores at individual sites.** | | |
| --- | --- | --- |
| **Beighton Maneuver** | **Right** | **Left** |
| **5^th^ finger** | 0.89 (0.79-0.99) | 0.81 (0.68-0.94) |
| **Thumb** | 1.00 | 1.00 |
| **Elbow** | 1.00 | 1.00 |
| **Knee** | 0.97 (0.92-1.00) | 0.95 (0.88-1.00) |
| **Trunk** | 0.92 (0.84-1.00) | |
